# Role of Escape Mutant-Specific T Cells in Suppression of HIV-1 Replication and Coevolution with HIV-1

**DOI:** 10.1128/JVI.01151-20

**Published:** 2020-09-15

**Authors:** Yu Zhang, Nozomi Kuse, Tomohiro Akahoshi, Takayuki Chikata, Hiroyuki Gatanaga, Shinichi Oka, Hayato Murakoshi, Masafumi Takiguchi

**Affiliations:** a Division of International Collaboration Research, Joint Research Center for Human Retrovirus Infection, Kumamoto University, Kumamoto, Japan; b Center for AIDS Research, Kumamoto University, Kumamoto, Japan; c AIDS Clinical Center, National Center for Global Health and Medicine, Tokyo, Japan; Emory University

**Keywords:** CTL, HIV-1, HLA-B*52:01, coevolution, escape mutation

## Abstract

The accumulation of HIV-1 escape mutations affects HIV-1 control by HIV-1-specific T cells. Some of these mutations can elicit escape mutant-specific T cells, but it still remains unclear whether they can suppress the replication of HIV-1 mutants. It is known that HLA-B*52:01-restricted RI8 (Gag 275 to 282; RMYSPTSI) is a protective T cell epitope in HIV-1 subtype B-infected Japanese individuals, though 3 Gag280A/S/V mutations are found in 26% of them. Gag280S and Gag280A were HLA-B*52:01-associated mutations, whereas Gag280V was not, implying a different mechanism for the accumulation of Gag280 mutations. In this study, we investigated the coevolution of HIV-1 with RI8-specific T cells and suppression of HIV-1 replication by its escape mutant-specific T cells both *in vitro* and *in vivo*. HLA-B*52:01^+^ individuals infected with Gag280A/S mutant viruses failed to elicit these mutant epitope-specific T cells, whereas those with the Gag280V mutant one effectively elicited RI8-6V mutant-specific T cells. These RI8-6V-specific T cells suppressed the replication of Gag280V virus and selected wild-type virus, suggesting a mechanism affording no accumulation of the Gag280V mutation in the HLA-B*52:01^+^ individuals. The responders to wild-type (RI8-6T) and RI8-6V mutant peptides had significantly higher CD4 counts than nonresponders, indicating that the existence of not only RI8-6T-specific T cells but also RI8-6V-specific ones was associated with a good clinical outcome. The present study clarified the role of escape mutant-specific T cells in HIV-1 evolution and in the control of HIV-1.

**IMPORTANCE** Escape mutant-specific CD8^+^ T cells were elicited in some individuals infected with escape mutants, but it is still unknown whether these CD8^+^ T cells can suppress HIV-1 replication. We clarified that Gag280V mutation were selected by HLA-B*52:01-restricted CD8^+^ T cells specific for the GagRI8 protective epitope, whereas the Gag280V virus could frequently elicit GagRI8-6V mutant-specific CD8^+^ T cells. GagRI8-6V mutant-specific T cells had a strong ability to suppress the replication of the Gag280V mutant virus both *in vitro* and *in vivo*. In addition, these T cells contributed to the selection of wild-type virus in HLA-B*52:01^+^ Japanese individuals. We for the first time demonstrated that escape mutant-specific CD8^+^ T cells can suppress HIV-1 replication and play an important role in the coevolution with HIV-1. Thus, the present study highlighted an important role of escape mutant-specific T cells in the control of HIV-1 and coevolution with HIV-1.

## Abstract (precis)

Escape mutant-specific CD8^+^ T cells were elicited in some individuals infected with escape mutants, but it is still unknown whether these CD8^+^ T cells can suppress HIV-1 replication. We clarified that Gag280V mutation were selected by HLA-B*52:01-restricted CD8^+^ T cells specific for the GagRI8 protective epitope, whereas the Gag280V virus could frequently elicit GagRI8-6V mutant-specific CD8^+^ T cells. GagRI8-6V mutant-specific T cells had a strong ability to suppress the replication of the Gag280V mutant virus both *in vitro* and *in vivo*. In addition, these T cells contributed to the selection of wild-type virus in HLA-B*52:01^+^ Japanese individuals. We for the first time demonstrated that escape mutant-specific CD8^+^ T cells can suppress HIV-1 replication and play an important role in the coevolution with HIV-1. Thus, the present study highlighted an important role of escape mutant-specific T cells in the control of HIV-1 and coevolution with HIV-1.

## INTRODUCTION

It is well known that HIV-1-specific CD8^+^ T cells play an important role in the control of HIV-1 (1–9). These T cells are considered to work as effector/memory T cells in therapeutic and prophylactic AIDS vaccines. The so-called “kick-and-kill” treatment, which combines latency-reversing agents with cytotoxic T lymphocytes (CTLs) or NK cells, is proposed to eradicate latent HIV-1 reservoirs from antiretroviral therapy (ART)-treated individuals (10, 11). A previous study in a nonhuman primate model of simian immunodeficiency virus showed that mosaic vaccines in combination with an immune modulator Toll-like receptor 7 (TLR7) agonist improved virologic control and delayed viral rebound following discontinuation of antiretroviral therapy and that the breadth of cellular immune responses correlated inversely with set point viral loads and correlated directly with time to viral rebound (12), suggesting that effective cellular immunity is required in kick-and-kill treatment. A recent clinical trial of a therapeutic vaccine in 26 ART-suppressed HIV-infected individuals who had started with ART during an acute infection demonstrated that the mosaic vaccine induced high levels of polyfunctional CD4^+^ T cells and CD8^+^ T cells, as well as Env-specific antibodies, but the effect of this vaccine to delay viral rebound following discontinuation of antiretroviral therapy was small compared to that of placebo controls (13).

HIV-1-specifc T cells select HIV-1 escape mutants affecting T cell recognition (14–17). Therefore, the existence of escape mutations in reservoir viruses and circulating viruses is a critical barrier for the eradication of latent HIV-1 reservoirs and prevention of HIV-1 infections. These escape mutant viruses can elicit mutant-specific T cells in some cases (18–21). A recent study showed that the transmission of human leukocyte antigen (HLA)-adapted mutations affects the clinical outcome in the acute phase of an HIV-1 infection (22). T cell responses to epitopes including HLA-adapted mutations are frequently detected in HIV-1 chronic infections (23), whereas they are rarely found in acute infections (22). Although some HLA-adapted mutations are known to be escape ones, it remains unknown whether T cells specific for epitopes having HLA-adapted or escape mutations can effectively suppress HIV-1 replication in chronic infections. Previous studies demonstrated that escape mutant-specific T cells fail to suppress replication of the mutant virus *in vitro* (19, 24, 25).

HLA class I alleles or haplotypes have consistently been shown to have a significant impact on the rate of HIV-1 disease progression to AIDS (26–34). HLA-B*57, HLA-B*27, and HLA-B*52 are associated with a slow progression to AIDS (26, 27, 31, 33–36), whereas HLA-B*35, HLA-B*58:02, and HLA-A*29:01-B*07:05-C*15:05 are associated with a rapid progression (28, 32, 34, 37–40). Whole-genome association analyses confirmed that HLA-B*57 and HLA-B*52:01 are the first and second strongest protective alleles, respectively, in Caucasian and/or African individuals (33, 40). A previous study demonstrated that HLA-B*52:01-C*12:02 is a protective haplotype in Japan, where HLA-B*57 and HLA-B*27 are very rare (31, 41). HLA-B*52:01 is found in more than 20% of Japanese individuals and is an allele with a relatively high frequency in East Asian countries, whereas it is detected in only 2% to 3% of Caucasians and is very rare in Africa (42, 43). Therefore, HLA-B*52:01-restricted immune responses to HIV-1 play an important role in HIV-1 control in Japanese and East Asian individuals more than in other ethnic groups (6, 44).

Recent studies on HIV-1 subtype B-infected Japanese individuals demonstrated that HLA-B*52:01-restricted HIV-1-specific CD8^+^ T cells for 4 epitopes (GagMI8 [Gag 198 to 205], GagWV8 [Gag 316 to 323], GagRI8 [Gag 275 to 282], and PolSI8 [Pol 654 to 661]) have the ability to suppress HIV-1 replication both *in vivo* and *in vitro* (6, 44). Of these epitopes, GagMI8, GagWV8, and PolSI8 are conserved ones among the subtype B viruses, whereas GagRI8 has 3 substitutions at Gag280 (Gag280S, Gag280A, and Gag280V) in 26% of HIV-1 subtype B-infected Japanese individuals (6, 45). A previous study on HLA-associated HIV-1 polymorphisms in HIV-1 subtype B-infected Japanese individuals showed that Gag280S and Gag280A accumulate in HLA-B*52:01^+^ individuals, whereas Gag280V do not (46), suggesting that Gag280S and Gag280A are escape mutations selected by HLA-B*52:01-restricted RI8-specific T cells. However, it is unknown whether Gag280V is an escape mutant or not and why RI8 is a protective epitope even though 26% of circulating viruses have these mutations.

In the present study, we investigated the mechanisms for the selection and accumulation of escape mutations at Gag280 in HIV-1 subtype B-infected Japanese individuals and for elicitation of escape mutant-specific T cells. Furthermore, we investigated the role of HLA-B*52:01-restricted T cells specific for the RI8 epitope or its mutants in the clinical outcome of Japanese individuals.

## RESULTS

### Selection and accumulation of Gag280S/A mutant viruses in HIV-1-infected HLA-B*52:01^+^ individuals.

**FIG 1:**
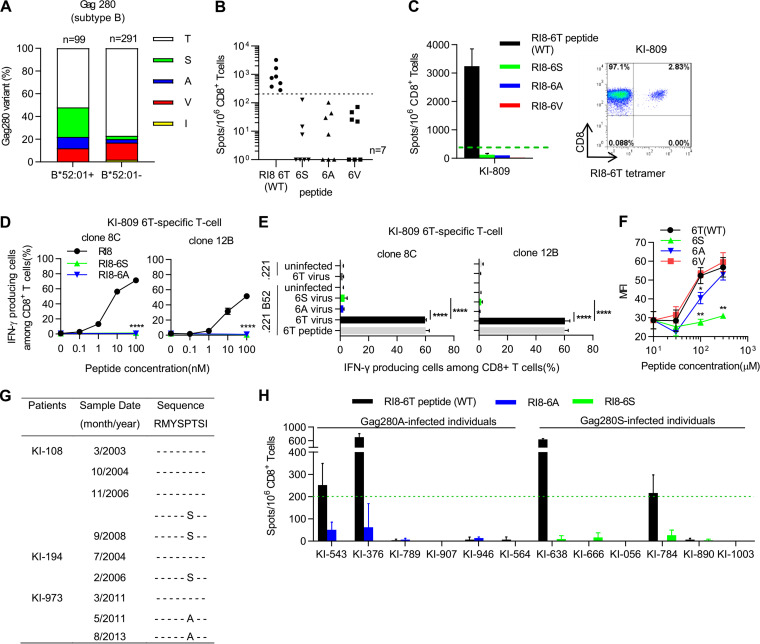
Recognition of RI8-6S and RI8-6A mutant epitopes by HLA-B*52:01-restricted RI8-6T-specific T cells. (A) Association of HLA-B*52:01 with mutations at Gag280 in 390 HIV-1 subtype B-infected Japanese individuals. Gag280S, Gag280A, Gag280V, and Gag280T were found in 26, 10, 12, and 51 HLA-B*52:01^+^ individuals, respectively; Gag280S, Gag280A, Gag280V, Gag280I, and Gag280T in were found 8, 8, 45, 6, and 224 HLA-B*52:01^−^ individuals, respectively. (B) T cell responses to RI8-6T peptide or its mutants. (C) Response (left) and identification (right) of RI8-specific T cells in PBMCs from KI-809 were analyzed by using the ELISPOT assay and RI8-6T tetramer, respectively. (D and E) Recognition of RI8-6S or -6A mutant epitopes by RI8-6T-specific T cell clones. T cell responses to 721.221-B*52:01 cells prepulsed with RI8-6T, RI8-6S, or RI8-6A peptide at various concentrations (D) and to those infected with NL43-Gag280T (wild-type), -Gag280S, or -Gag280A (E) were analyzed by performing ICS assays. The frequencies of p24 antigen-positive cells among 721.221-B*52:01 cells infected with NL43-Gag280T, -Gag280S, and -Gag280A and 721.221 cells infected NL43-Gag280T were 59.6%, 54.6%, 59%, and 59%, respectively (E). (F) Binding affinity of RI8 and its mutant peptides to HLA-B*52:01. (G) Longitudinal sequence analysis at Gag280 in 3 HLA-B*5201^+^ Japanese individuals. (H) T cell responses to RI8 and RI8-6A peptides in 6 Gag280A-infected and those to RI8 and RI8-6S in 6 Gag280S-infected individuals. Results are given as means with SD (*n* = 3). The dotted line indicates the threshold for a positive response (B, C, and H). Statistical analysis was performed by using the unpaired *t* test (D to F). *, *P* < 0.05; **, *P* < 0.01; ****, *P* < 0.0001. WT, wild type.

To investigate HLA-B*52:01-associated mutations at Gag280 in HIV-1 subtype B infections, we analyzed the sequences around this position from 390 treatment-naive Japanese individuals chronically infected with HIV-1 subtype B (99 HLA-B*52:01^+^ and 291 HLA-B*52:01^−^ ones). The frequencies of Gag280S and Gag280A mutants were significantly higher in the HLA-B*52:01^+^ individuals than in the HLA-B*52:01^−^ ones (*P* = 6.74E^−11^ and *q* = 3.37E^−10^ and *P* = 0.00837 and *q* = 0.0140, respectively), whereas no significant difference was observed in the frequency of Gag280V mutants between HLA-B*52:01^+^ and HLA-B*52:01^−^ individuals (Fig. 1A). These results indicate that Gag280S and Gag280A mutants had accumulated in the HLA-B*52:01^+^ individuals but that Gag280V ones had not.

A previous study revealed that T cell responses to 3 mutant epitopes, RI8-6S, -6A, and -6V, were not detectable in 3 HLA-B*52:01^+^ individuals having T cells specific for the RI8-6T (RMYSPTSI) wild-type epitope (6). To confirm this result, we analyzed an additional 7 HLA-B*52:01^+^ individuals infected with Gag280T wild-type virus who had RI8-6T-specific T cells. T cell responses to RI8-6S, -6A, or -6V mutant peptides were not found in these individuals (Fig. 1B), indicating that these mutant epitopes could not be recognized by RI8-6T-specific T cells. To investigate in detail the recognition of RI8-6T-specific T cells for these mutant epitopes, we established RI8-6T-specific T cell clones from wild-type virus-infected individual KI-809, who had a strong T cell response to the RI8-6T peptide (Fig. 1C, left) and a high number of RI8-6T-HLA-B*52:01 tetramer-binding T cells (Fig. 1C, right). Two RI8-6T-specific T cell clones failed to recognize not only 721.221 cells expressing HLA-B*52:01 (721.221-B*52:01) prepulsed with RI8-6S or RI8-6A mutant peptide (Fig. 1D) but also those infected with these mutant viruses (Fig. 1E). An HLA class I stabilization assay using RMA-S-B*52:01 cells revealed that the binding affinity of RI8-6S and RI8-6A peptides for HLA-B*52:01 molecules was weaker than that of the RI8-6T peptide, though that of the RI8-6S peptide was much weaker than that of the RI8-6A one (Fig. 1F). These findings together suggest that the Gag280S mutation critically affected the epitope presentation in the cells infected with the Gag280S mutant virus and that the Gag280A mutation may have affected T cell receptor (TCR) recognition rather than the presentation of the epitope.

**FIG 2:**
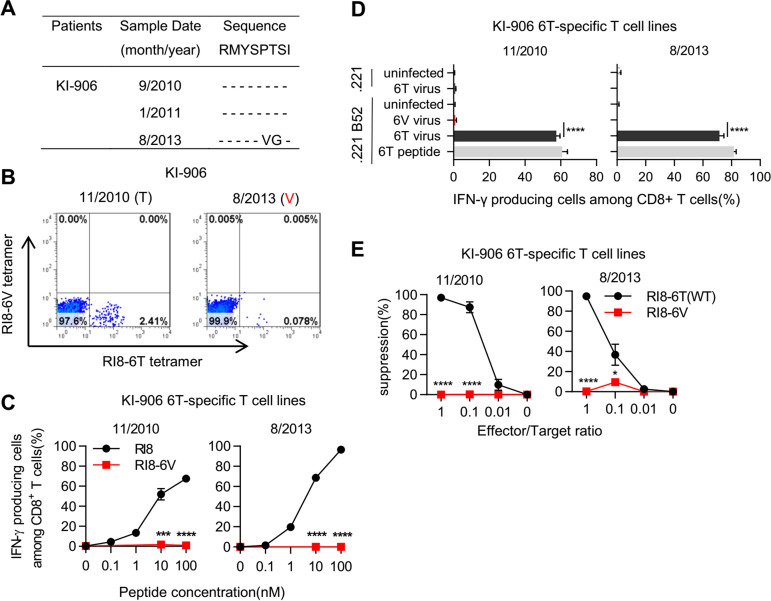
Recognition of RI8-6V mutant epitope by HLA-B*52:01-restricted RI8-6T-specific T cells. (A) Longitudinal sequence analysis at Gag280 in an HLA-B*5201^+^ Japanese individual. (B) Identification of RI8-specific T cells among PBMCs from KI-906 before and after the emergence of the Gag280V mutant virus. (C and D) Recognition of RI8-6V mutant epitope by RI8-6T-specific T cell lines. T cell responses to 721.221-B*52:01 cells prepulsed with RI8-6T or -6V peptide (C) and to those infected with NL43-Gag280T or -Gag280V (D) were analyzed. Frequencies of p24-positive cells among 721.221-B*52:01 cells infected with NL43-Gag280T and -Gag280V and 721.221 cells infected with -Gag280T were 60.8%, 58.0%, and 66.0%, respectively (D). (E) Ability of RI8-6T-specific T cells to suppress the replication of Gag280T and Gag280V virus. Results are given as means with SDs (*n* = 3). Statistical analysis was performed by using the unpaired *t* test (B to D). *, *P* < 0.05; ***, *P* < 0.001; ****, *P* < 0.0001.

We performed a longitudinal sequence analysis at Gag280 in 13 HLA-B*52:01^+^ individuals infected with the wild-type virus at the first sampling. The results demonstrated the T-to-S substitution (KI-108 and KI-194), the T-to-A substitution (KI-973), and the T-to-V substitution (KI-906) in 4 of them after the second sampling (Fig. 1G and Fig. 2A). These results support the idea that these mutations were selected by RI8-6T-specific T cells and accumulated in the HLA-B*52:01^+^ individuals.

We further investigated whether RI8-6A- or RI8-6S-specific T cells were elicited in HLA-B*52:01^+^ individuals infected with Gag280A or Gag280S virus by performing the enzyme-linked immunosorbent spot assay (ELISPOT) assay. The results showed that these mutant epitope-specific T cells were not elicited in them (Fig. 1H). Thus, these mutations critically affected the elicitation of these mutant epitope-specific T cells *in vivo*.

### Selection of Gag280V mutant virus by RI8-6T-specific CD8^+^ T cells.

T cell responses to the 6V mutant were not found in 7 HLA-B*52:01^+^ individuals who were infected with the wild-type virus and had RI8-6T-specific T cells (Fig. 1B), suggesting that RI8-6T-specific T cells could not recognize the RI8-6V mutant. To clarify the ability of RI8-6T-specific T cells to recognize the mutant, we investigated RI8-6T-specific T cells in individual KI-906, who was infected with the Gag280T virus before January 2011, followed by the emergence of the Gag280V mutant in August 2013 (Fig. 2A). Flow cytometric analysis using RI8-6T- and RI8-6V-HLA-B*52:01 tetramers revealed that KI-906 had RI8-6T-specific T cells as 2.41% of total CD8^+^ T cells in November 2010 but as only 0.08% of them in August 2013 (Fig. 2B). We established RI8-6T-specific T cell lines from peripheral blood mononuclear cells (PBMCs) at these time points by sorting for RI8-6T-specific T cells and analyzed the ability of these T cell lines to recognize target cells prepulsed with RI8-6V peptide and those infected with Gag280-6V virus. Two RI8-6T-specific T cell lines recognized 721.221-B*52:01 cells prepulsed with RI8-6T peptide and those infected with Gag280-6T virus, whereas they failed to recognize those prepulsed with RI8-6V peptide and those infected with Gag280-6V virus (Fig. 2C and D). In addition, a viral suppression assay showed that these T cell lines strongly suppressed the replication of the Gag280T virus but not that of the Gag280-6V one (Fig. 2E). Taken together, these results support the idea that the Gag280V mutation could be selected by RI8-6T-specific T cells. However, it remains unclear as to why the Gag280V mutant did not accumulate in the subtype B-infected HLA-B*52:01^+^ individuals.

### Selection of Gag280T wild-type virus by RI8-6V-specific and cross-reactive T cells.

**FIG 3:**
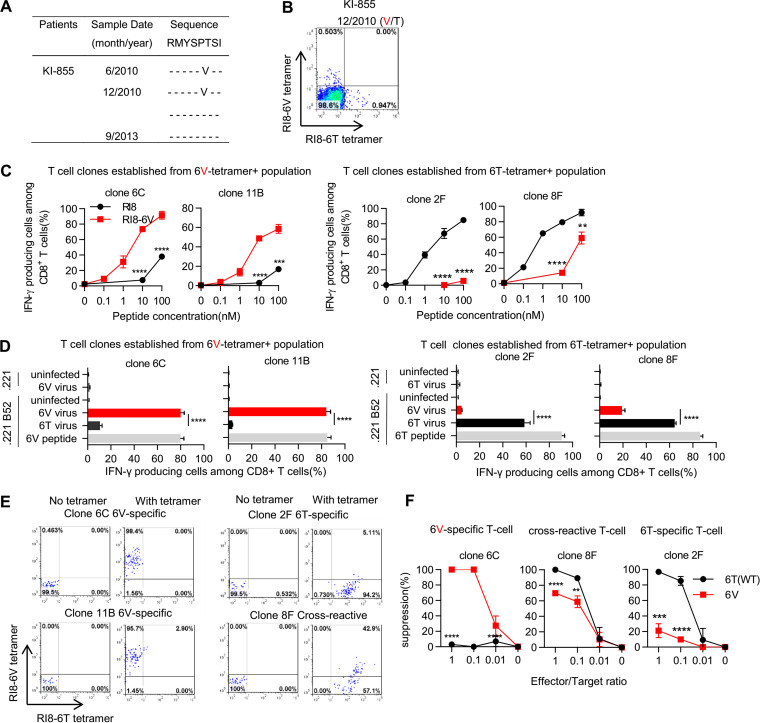
Ability of RI8-6V-specific T cells to recognize RI8-6V-infected cells and to suppress RI8-6V replication. (A) Longitudinal sequence analysis at Gag280 in an HLA-B*5201^+^ Japanese individual infected with HIV-1 subtype B. (B) Identification by tetramer staining of RI8-specific T cells in PBMCs from KI-855 infected with a mixture of Gag280T and Gag280V viruses in December 2010. (C and D) Recognition of RI8-6T or -6V epitope by T cell clones established from the RI8-6V tetramer^+^ or RI8-6T tetramer^+^ T cell population. Responses of these clones to 721.221-B*52:01 cells prepulsed with RI8-6T or -6V peptide (C) and to those infected with NL43-Gag280T or -Gag280V were analyzed by using the ICS assay (D). The frequencies of p24 antigen-positive cells among 721.221-B*52:01 cells infected with NL43-Gag280T and Gag280V were 35.3% and 28.9%, respectively, whereas those of 721.221 infected with NL43-Gag280T and -Gag280V were 30.3% and 34.8%, respectively (D). (E) Staining of RI8-6V-specific, RI8-6T-specific, and cross-reactive T cell clones with both HLA-B*52:01-RI8-6T and HLA-B*52:01-RI8-6V tetramers. (F) Ability of RI8-6V-specific, cross-reactive, and RI8-6T-specific T cell clones to suppress the replication of Gag280-6T and -6V viruses. Results are given as means with SD (*n* = 3). percent suppression of HIV-1 replication is presented. Statistical analysis was performed by using the unpaired *t* test. **, *P* < 0.01; ***, *P* < 0.001; ****, *P* < 0.0001 (C, D, and F).

We performed a longitudinal analysis at Gag280 in 5 HLA-B*52:01^+^ individuals infected with the Gag280V virus at the first sampling and found that HLA-B*52:01^+^ individual KI-855 had been infected with the Gag280V virus in June 2010 and then showed the presence of both Gag280V and Gag280T viruses in December 2010, and finally that of only the Gag280T virus in September 2013 (Fig. 3A). Analysis using RI8-6T- or RI8-6V-HLA-B*52:01 tetramers demonstrated the existence of both RI8-6T-specific and RI8-6V-specific HLA-B*52:01-restricted T cells in PBMCs collected from KI-855 in December 2010 (Fig. 3B). To investigate these T cells, we established RI8-6T-specific and RI8-6V-specific T cell clones from this individual’s PBMCs collected. Two RI8-6V-specific T cell clones, 6C and 11B, had a strong ability to recognize 721.221-B*52:01 cells prepulsed with RI8-6V peptide (Fig. 3C) and those infected with the Gag280V virus (Fig. 3D). On the other hand, 2 RI8-6T-specific T cell clones, 2F and 8F, effectively recognized 721.221-B*52:01 cells prepulsed with RI8-6T peptide, though the latter one had a weak ability to cross-recognize those prepulsed with RI8-6V peptide at a high concentration (Fig. 3C). Both clones effectively recognized 721.221-B*52:01 cells infected with the Gag280T virus, whereas clone 2F and clone 8F failed to recognize and weakly recognized, respectively, those infected with the Gag280V virus (Fig. 3D). Analysis using the B*52:01 tetramers demonstrated that clone 2F and clones 6C and 11B were RI8-6T-specific and RI8-6V-specific T cells, respectively, and that clone 8F was cross-reactive T cells that strongly bound to the RI8-6T tetramer but weakly to the RI8-6V one (Fig. 3E). Thus, 3 types of RI8-specific T cells (RI8-6V-specific, RI8-6T-specific, and cross-reactive T cells) were elicited in KI-855. Further analyses using viral suppression assays demonstrated that the RI8-6V-specific T cell clone effectively suppressed the replication of the Gag280V virus but not that of the Gag280T one and that the RI8-6T-specific T cell clone suppressed the replication of the Gag280-6T virus but not that of the Gag280-6V one (Fig. 3F). The cross-reactive T cell clone revealed a strong ability to suppress the replication of both viruses, though the viral suppression ability for the Gag280-6V virus was weaker than that for the Gag280-6T one (Fig. 3F). These results indicate that T cells having a strong ability to suppress the replication of the Gag280V virus were elicited in HLA-B*5201^+^ individuals infected with the Gag280V virus. KI-855 revealed a reversion of Gag280V to Gag280T after the elicitation of RI8-6V-specific and cross-reactive T cells. This finding supports the idea that RI8-6V-specific T cells and/or cross-reactive T cells selected the wild-type virus.

**FIG 4:**
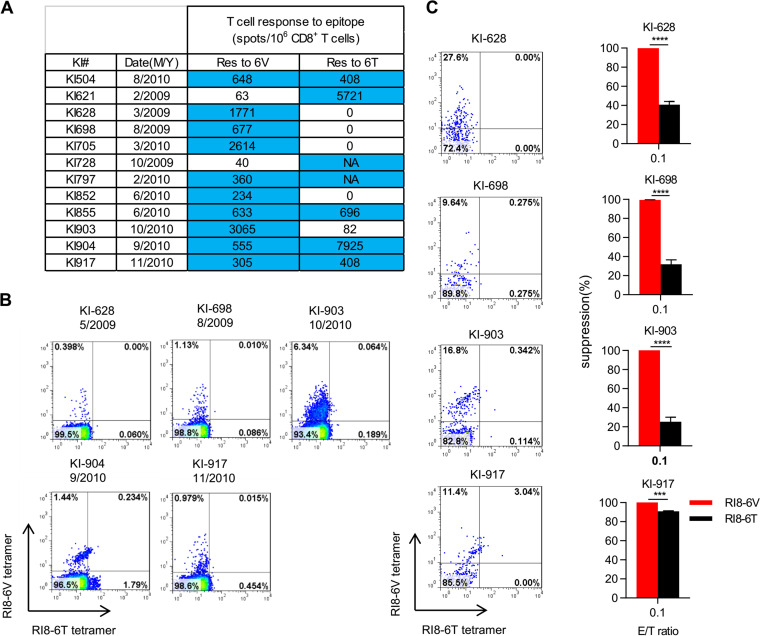
Detection of RI8-6V-specific T cells and their ability to suppress RI8-6V replication in Gag280V-infected HLA-B*5201^+^ Japanese individuals. (A) T cell responses to RI8-6V (6V) or RI8-6T (6T) epitopes were analyzed by performing the IFN-γ ELISPOT assay. The blue shading indicates a positive response in the ELISPOT assay (>200 spots/10^6^ CD8^+^ T cells). NA, not analyzed. (B) Identification by tetramer staining of RI8-specific T cells among PBMCs from 5 Gag280V-infected individuals. PBMCs were stained with HLA-B*52:01-RI8-6T and HLA-B*52:01-RI8-6V tetramers. (C) Ability of RI8-6V-specific T cells to suppress the replication of Gag280-6T and -6V viruses. RI8-6V-specific T cell lines were induced from PBMCs of 4 individuals by stimulating the PBMCs with RI8-6V peptide and culturing them for 14 days. The frequencies of RI8-6V-specific and RI8-6T-specific T cells were measured by staining with both HLA-B*52:01-RI8-6T and HLA-B*52:01-RI8-6V tetramers (left). Activated CD4^+^ T cells from an HLA-B*52:01^+^ individual infected with NL43-Gag280T or -Gag280V were cocultured with RI8-6V-specific T cells at an E:T ratio of 0.1:1 (right). Results are given as means with SD (*n* = 3). Percent suppression of HIV-1 replication is presented. Statistical analysis was performed by using the unpaired *t* test. ***, *P* < 0.001; ****, *P* < 0.0001.

We next analyzed RI8-6V-specific and/or cross-reactive T cells in all 12 HLA-B*5201^+^ individuals infected with the Gag280V virus. RI8-6V-specific T cells were detected in 10 of these individuals, though RI8-6T-specific T cells were also found in 4 of them (Fig. 4A). The analysis of PBMCs from 5 individuals, performed by using specific tetramers, confirmed the existence of RI8-6V-specific T cells in these 5 individuals (Fig. 4B). RI8-6V-specific T cell lines established from 4 individuals demonstrated a strong ability to suppress replication of Gag280V mutant virus, though those from KI-917 exhibited a strong ability to suppress the replication of both viruses (Fig. 4C). These results demonstrated that RI8-6V-specific T cells and/or cross-reactive T cells were frequently elicited in HLA-B*5201^+^ individuals infected with Gag280V virus and that these T cells could suppress the replication of the Gag280V mutant virus.

### Contribution of RI8-6T and RI8-6V-specific CD8^+^ T cells to control of HIV-1 in subtype B infection.

**FIG 5:**
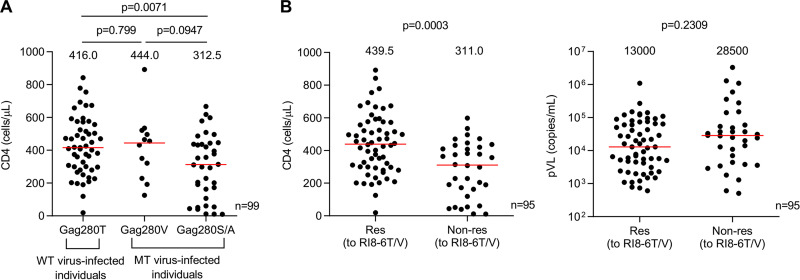
Comparison of clinical outcomes among individuals infected with HIV-1 having different Gag 280 mutations and between T cell responders to RI8 and nonresponders. (A) Comparison of CD4 counts for individuals infected with Gag280T (wild type), Gag280V, or Gag280S/Gag280A virus among 99 subtype B-infected HLA-B*52:01^+^ Japanese individuals. (B) Association of T cell responses to RI8-6T/6V with clinical outcome. Comparison of CD4 count and pVL between T cell responders and nonresponders to RI8-6T/6V among 95 subtype B-infected HLA-B*52:01^+^ Japanese individuals is shown. Statistical analysis was performed by using the Mann-Whitney test. The value indicated by the red line in each graph represents the median of the CD4 count.

Next, we analyzed the effect of Gag280 mutations on the clinical outcome in subtype B-infected HLA-B*52:01^+^ Japanese individuals. The individuals infected with the Gag280T virus had significantly higher CD4 counts than those with Gag280S/A virus, whereas the Gag280V-infected individuals showed a trend for a higher CD4 count than the Gag280S/A-infected ones (Fig. 5A). These results suggest that RI8-6T/6V-specific T cells may have suppressed the replication of HIV-1 in these individuals. We therefore investigated the association of T cell responses to RI8-6T/6V with the clinical outcome. Responders to RI8-6T or 6V peptide showed significantly higher CD4 counts and trends toward a lower plasma viral load (pVL) than nonresponders (Fig. 5B), indicating that both RI8-6T-specific and RI8-6V-specific T cells contributed to the suppression of HIV-1 replication in subtype B-infected HLA-B*52:01^+^ Japanese individuals.

## DISCUSSION

A previous study on HLA-associated HIV-1 polymorphisms in HIV-1 subtype B-infected Japanese individuals showed that Gag280S and Gag280A are HLA-B*52:01-associated mutations, whereas Gag280V is not (46). This finding suggested that Gag280S and Gag280A, but not Gag280V, are escape mutations selected by HLA-B*52:01-restricted RI8-specific T cells. However, the present study clearly demonstrated that HLA-B*52:01-restricted RI8-specific T cells failed to recognize cells infected with Gag280V, Gag280A, or Gag280S mutant virus, indicating that these mutations were escape ones. Gag280V had not accumulated in the HLA-B*52:01^+^ individuals, whereas this mutation was found more frequently than the Gag280A or Gag280S mutation in Japanese individuals. These findings together suggested the presence of a mechanism responsible for the absence of accumulation of the Gag280V mutation in the HLA-B*52:01^+^ individuals. Our hypothesis is that RI8-6V-specific T cells were elicited in HLA-B*52:01^+^ individuals infected with the Gag280V mutant virus and that these T cells selected the wild-type virus. Indeed, we demonstrated that RI8-6V-specific T cells were detected in most of the HLA-B*52:01^+^ individuals infected with Gag280V mutant virus and that these T cells had a strong ability to suppress replication of the Gag280V mutant virus.

The results of an HLA class I stabilization assay showed that the binding affinity of RI8-6S peptide for HLA-B*52:01 molecules was much weaker than that of the RI8-6T peptide but that the affinity of the RI8-6V peptide was identical to that of the RI8-6T. These findings together suggest that position 6 is a critical site for both the peptide binding to HLA-B*52:01 and TCR recognition, though this position is not an anchor residue (47). The affinity of the RI8-6S peptide was much weaker than that of the RI8-6T or the RI8-6V one, suggesting that the RI8-6S epitope peptide could not be presented in the cells infected with the Gag280S mutant virus. On the other hand, the Gag280A mutation weakly affected the peptide binding affinity, suggesting this mutation may have affected TCR recognition. RI8-6T-specific T cells failed to recognize the RI8-6V epitope, whereas RI8-6V-specific T cells were elicited in the individuals infected with Gag280V mutant virus. These findings suggest that there were 2 T cell repertoires for RI8 in HLA-B*52:01^+^ individuals, one having high-affinity TCRs for RI8-6T and the other for RI8-6V.

HLA-B*52:01 is protective allele in the subtype B and C infections (31, 33, 40), whereas Gag RI8 is one of protective T cell epitopes restricted by HLA-B*52:01 (6). RI8-6T-specific T cells failed to recognize the cells infected with Gag280S/A mutant viruses, and T cells specific for RI8-6A/6S mutant epitopes were not elicited in the individuals infected with these viruses (Fig. 1H). These findings suggest that the accumulation of Gag280S/A mutations would critically affect suppression of HIV-1 replication by these specific T cells *in vivo*. Indeed, HLA-B*52:01^+^ Japanese individuals infected with Gag280S/A mutant viruses had significantly lower CD4 counts than those infected with the wild-type virus. In contrast, RI8-6V-specific T cells, which were frequently elicited in Gag280V virus-infected HLA-B*52:01^+^ individuals, had a strong ability to suppress replication of Gag280V mutant viruses *in vitro*. Indeed, our analysis showed that no significant difference in CD4 count was found between individuals infected with Gag280T virus and those with the Gag280V one, suggesting that the Gag280V mutation did not affect the control of HIV-1. Since the accumulation of Gag280S/A mutations was found in only 20% of the HLA-B*52:01^+^ individuals, GagRI8 is still a protective T cell epitope in them.

Three of 4 HLA-B*52:01-restricted epitopes are conserved among circulating HIV-1 subtype B viruses (6), and T cells specific for these epitopes have a strong ability to suppress HIV-1 replication *in vivo* (6, 44). These epitopes may be targets for prophylactic T cell vaccines and a cure for HIV-1. The wild-type sequence of RI8 is found in only 60% of Japanese individuals infected with the subtype B virus, suggesting that this epitope may not be useful for a T cell vaccine and AIDS cure. However, the Gag280V mutant virus could elicit RI8-6V mutant virus-specific T cells in individuals infected with this mutant virus, and these T cells could suppress replication of the mutant virus. Since approximately 80% of circulating viruses have Gag280T/V, chimeric antigens (Ags) containing both RI8-6T and RI8-6V epitopes could be useful for a vaccine and cure of AIDS. Thus, the present study showed that a T cell epitope including an escape mutation could be target for a T cell vaccine and AIDS cure. However, since it is still unknown whether other escape mutant epitopes also could elicit specific T cells that could effectively suppress HIV-1 mutant viruses, further studies on T cell recognition for escape HIV-1 mutants are required for generation of chimeric vaccine antigens that should contribute to the development of a prophylactic T cell vaccine and AIDS cure.

**FIG 6:**
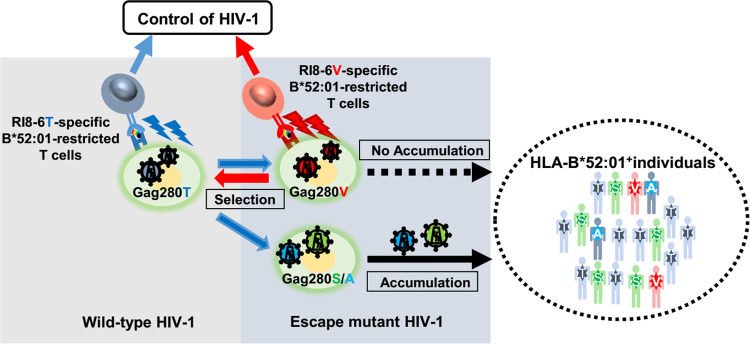
Summary of this study.

In the present study, we demonstrated a mechanism for the accumulation of different Gag280 mutations in subtype B-infected Japanese and for coevolution of HIV-1 with HIV-1-specific T cells as well as the important role of mutant specific T cells in the suppression of HIV-1 replication *in vivo* (Fig. 6). The results of the present study strongly impact our understanding of the role of mutant epitope-specific T cells in the control of HIV-1 and imply their potential usefulness for a prophylactic AIDS vaccine and AIDS cure.

## MATERIALS AND METHODS

### Subjects.

All treatment-naive Japanese individuals chronically infected with HIV-1 subtype B were recruited from the National Center for Global Health and Medicine, Japan. This study was approved by the ethics committees of Kumamoto University (RINRI-1340 and GENOME-342) and the National Center for Global Health and Medicine (NCGM-A-000172-01). Informed consent was obtained from all individuals according to the Declaration of Helsinki. Peripheral blood mononuclear cells (PBMCs) were separated from whole blood. HLA types of HIV-infected individuals were determined by standard sequence-based genotyping. The pVLs of the individuals at their first visit were measured by using the Cobas TaqMan HIV-1 real-time PCR version 2.0 assay (Roche Diagnostics, NJ).

### Cell lines.

C1R cells expressing HLA-B*52:01 (C1R-B*52:01), 721.221 cells expressing CD4 molecules and HLA-B*52:01 (721.221-B*52:01), and RMA-S cells expressing HLA-B*52:01 (RMA-S-B*52:01) were previously generated (47, 48). These cells were maintained in RPMI 1640 medium (Invitrogen) containing 5% fetal calf serum (FCS; R5) and 0.15 mg/ml of hygromycin B or 0.2 mg/ml of neomycin.

### HIV-1 mutant clones.

NL4-3 mutants (NL4-3-Gag280V, -Gag280-6S, and -Gag280-6A) were previously generated (6).

### Sequence of autologous virus.

Determination of the epitope sequence for RI8 was performed as previously described (46). The RI8 sequence data from 390 chronically HIV-1 subtype B-infected treatment-naive Japanese individuals were analyzed after excluding individuals having a mixture of amino acid sequences at Gag280 from previously analyzed ones (46) and adding new data from 16 individuals.

### IFN-γ ELISPOT assay.

*Ex vivo* gamma interferon (IFN-γ) ELISPOT assays were performed as previously described (6, 49). The number of spots for a T cell response to each peptide was finally calculated by subtracting the number of spots in wells without peptides. The mean + 3 standard deviations (SD) of the spot number of samples from 13 HIV-1 naive individuals for the peptides was 162 spots/10^6^ CD8^+^ T cells (6, 49). Therefore, we defined >200 spots/10^6^ CD8^+^ T cells as positive responses.

### Tetramer staining.

HLA-B*52:01-RI8-6T/6V tetrameric complexes (tetramers) were generated as previously described (50, 51). PBMCs or HIV-1-specific T cell clones/lines were stained with a combination of phycoerythrin (PE)-conjugated RI8-6T and allophycocyanin (APC)-conjugated RI8-6V-HLA-B*52:01 tetramers at 100 nM at 37°C for 30 min. The cells were subsequently stained with fluorescein isothiocyanate (FITC)-conjugated anti-CD3 (Dako, Glostrup, Denmark), Pacific blue-conjugated anti-CD8 monoclonal antibody (MAb) (BD Biosciences), and 7-aminoactinomycin D (7-AAD; BD Pharmingen) at 4°C for 30 min and analyzed with a FACSCanto II (BD Bioscience, CA). The frequency of HLA-tetramer^+^ cells was measured after gating of the CD3^+^ CD8^+^ population.

### Generation of epitope-specific T cell clones or lines.

PBMCs were stained with PE- or APC-conjugated tetramers, FITC-conjugated anti-CD3 (Dako, Glostrup, Denmark), Pacific blue-conjugated anti-CD8 MAb (BD Biosciences), and 7-AAD (BD Pharmingen), after which CD3^+^ CD8^+^ 7AAD^−^ tetramer^+^ T cells were sorted in U-bottomed 96-well microtiter plates (1 cell/well for T cell clones and 100 to 500 cells/well for T cell lines) by using a FACSAria (BD Biosciences). The sorted cells were stimulated with the corresponding epitope peptide and cultured as previously described (51). After 2 to 3 weeks in culture, epitope-specific CD8^+^ T cells were used in functional assays after their purity had been confirmed by flow cytometry analysis using tetramers.

### Intracellular cytokine staining (ICS) assay.

721.221 cells prepulsed with HIV-1 epitope peptide or 721.221 cells infected with HIV-1 were cocultured with T cell clones or lines in a 96-well plate for 2 h at 37°C. Brefeldin A (10 μg/ml) was then added, and the cells were incubated further for 4 h at 37°C. The cells were then fixed with 4% paraformaldehyde and incubated in permeabilization buffer (0.1% saponin–10% fetal bovine serum [FBS]–phosphate-buffered saline [PBS]), after which they were stained with APC-conjugated anti-CD8 MAb (Dako, Denmark) followed by FITC-conjugated anti-IFN-γ MAb (BD Biosciences). The percentage of IFN-γ-producing cells among the CD8^+^ T cell population was determined by use of the FACSCanto II.

### HLA stabilization assay.

The affinity of peptide binding to HLA-B*52:01 was examined by using RMA-S-B*52:01 cells as previously described (52, 53). Briefly, these RMA-S transfectant cells were cultured at 26°C for 16 h, then pulsed with peptides at 26°C for 1 h, and subsequently incubated at 37°C for 3 h. Staining of cell surface HLA molecules was performed by using anti-HLA class I α3 domain MAb TP25.99 (54) and FITC-conjugated sheep anti-mouse IgG (Jackson ImmunoResearch). The fluorescence intensity was measured with the FACSCanto II.

### HIV-1 replication suppression assay.

The ability of epitope-specific CD8^+^ T cells to suppress HIV-1 replication was measured as described previously (24, 55). CD4^+^ T cells isolated from HLA-matched healthy donor PBMCs were infected with HIV-1 virus and then cocultured with epitope-specific T cells at effector-to-target cell (E:T) ratios of 1:1, 0.1:1, and 0:1. When RI8-6V-specific bulk T cells were used as effector T cells (Fig. 4C), the number of effector T cells was calculated as a total number of T cells × percent RI8-6V tetramer^+^ T cells. On day 5 postinfection, the concentration of p24 antigen in the culture supernatant was measured by using an enzyme-linked immunosorbent assay (ELISA) kit (HIV-1 p24 Ag ELISA kit; ZeptoMetrix). The percent suppression was calculated as follows: (concentration of p24 without CTLs – concentration of p24 with CTLs)/concentration of p24 without CTLs × 100.

### Statistical analysis.

The frequency of the mutation between HLA^+^ and HLA^−^ individuals was statistically analyzed by using Fisher’s exact test. Groups were compared by performing the unpaired *t* test or two-tailed Mann-Whitney U test. *P* values of <0.05 were considered significant

## References

[1] Borrow P, Lewicki H, Hahn BH, Shaw GM, Oldstone MB 1994 Virus-specific CD8+ cytotoxic T-lymphocyte activity associated with control of viremia in primary human immunodeficiency virus type 1 infection. J Virol 68:6103–6110. doi:10.1128/JVI.68.9.6103-6110.1994. 8057491 PMC237022

[2] Koup RA, Safrit JT, Cao Y, Andrews CA, McLeod G, Borkowsky W, Farthing C, Ho DD 1994 Temporal association of cellular immune responses with the initial control of viremia in primary human immunodeficiency virus type 1 syndrome. J Virol 68:4650–4655. doi:10.1128/JVI.68.7.4650-4655.1994. 8207839 PMC236393

[3] Ogg GS, Jin X, Bonhoeffer S, Dunbar PR, Nowak MA, Monard S, Segal JP, Cao Y, Rowland-Jones SL, Cerundolo V, Hurley A, Markowitz M, Ho DD, Nixon DF, McMichael AJ 1998 Quantitation of HIV-1-specific cytotoxic T lymphocytes and plasma load of viral RNA. Science 279:2103–2106. doi:10.1126/science.279.5359.2103. 9516110

[4] Appay V, Douek DC, Price DA 2008 CD8+ T cell efficacy in vaccination and disease. Nat Med 14:623–628. doi:10.1038/nm.f.1774. 18535580

[5] Jones RB, Walker BD 2016 HIV-specific CD8^+^ T cells and HIV eradication. J Clin Invest 126:455–463. doi:10.1172/JCI80566. 26731469 PMC4731167

[6] Murakoshi H, Akahoshi T, Koyanagi M, Chikata T, Naruto T, Maruyama R, Tamura Y, Ishizuka N, Gatanaga H, Oka S, Takiguchi M 2015 Clinical control of HIV-1 by cytotoxic T cells specific for multiple conserved epitopes. J Virol 89:5330–5339. doi:10.1128/JVI.00020-15. 25741000 PMC4442500

[7] Collins DR, Gaiha GD, Walker BD 12 2 2020 CD8 T cells in HIV control, cure and prevention. Nat Rev Immunol doi:10.1038/s41577-020-0274-9. PMC7222980 32051540

[8] Murakoshi H, Zou C, Kuse N, Akahoshi T, Chikata T, Gatanaga H, Oka S, Hanke T, Takiguchi M 2018 CD8 T cells specific for conserved, cross-reactive Gag epitopes with strong ability to suppress HIV-1 replication. Retrovirology 15:46. doi:10.1186/s12977-018-0429-y. 29970102 PMC6029025

[9] Takata H, Buranapraditkun S, Kessing C, Fletcher JLK, Muir R, Tardif V, Cartwright P, Vandergeeten C, Bakeman W, Nichols CN, Pinyakorn S, Hansasuta P, Kroon E, Chalermchai T, O’Connell R, Kim J, Phanuphak N, Robb ML, Michael NL, Chomont N, Haddad EK, Ananworanich J, Trautmann L, RV254/SEARCH010 and the RV304/SEARCH013 Study Groups. 2017 Delayed differentiation of potent effector CD8 T cells reducing viremia and reservoir seeding in acute HIV infection. Sci Transl Med 9:eaag1809. doi:10.1126/scitranslmed.aag1809. 28202771 PMC5678930

[10] Sengupta S, Siliciano RF 2018 Targeting the latent reservoir for HIV-1. Immunity 48:872–895. doi:10.1016/j.immuni.2018.04.030. 29768175 PMC6196732

[11] Barouch DH, Deeks SG 2014 Immunologic strategies for HIV-1 remission and eradication. Science 345:169–174. doi:10.1126/science.1255512. 25013067 PMC4096716

[12] Borducchi EN, Cabral C, Stephenson KE, Liu J, Abbink P, Ng'ang'a D, Nkolola JP, Brinkman AL, Peter L, Lee BC, Jimenez J, Jetton D, Mondesir J, Mojta S, Chandrashekar A, Molloy K, Alter G, Gerold JM, Hill AL, Lewis MG, Pau MG, Schuitemaker H, Hesselgesser J, Geleziunas R, Kim JH, Robb ML, Michael NL, Barouch DH 2016 Ad26/MVA therapeutic vaccination with TLR7 stimulation in SIV-infected rhesus monkeys. Nature 540:284–287. doi:10.1038/nature20583. 27841870 PMC5145754

[13] Colby DJ, Sarnecki M, Barouch DH, Tipsuk S, Stieh DJ, Kroon E, Schuetz A, Intasan J, Sacdalan C, Pinyakorn S, Grandin P, Song H, Tovanabutra S, Shubin Z, Kim D, Paquin-Proulx D, Eller MA, Thomas R, de Souza M, Wieczorek L, Polonis VR, Pagliuzza A, Chomont N, Peter L, Nkolola JP, Vingerhoets J, Truyers C, Pau MG, Schuitemaker H, Phanuphak N, Michael N, Robb ML, Tomaka FL, Ananworanich J 2020 Safety and immunogenicity of Ad26 and MVA vaccines in acutely treated HIV and effect on viral rebound after antiretroviral therapy interruption. Nat Med 26:498–501. doi:10.1038/s41591-020-0774-y. 32235883

[14] Kawashima Y, Pfafferott K, Frater J, Matthews P, Payne R, Addo M, Gatanaga H, Fujiwara M, Hachiya A, Koizumi H, Kuse N, Oka S, Duda A, Prendergast A, Crawford H, Leslie A, Brumme Z, Brumme C, Allen T, Brander C, Kaslow R, Tang J, Hunter E, Allen S, Mulenga J, Branch S, Roach T, John M, Mallal S, Ogwu A, Shapiro R, Prado JG, Fidler S, Weber J, Pybus OG, Klenerman P, Ndung’u T, Phillips R, Heckerman D, Harrigan PR, Walker BD, Takiguchi M, Goulder P 2009 Adaptation of HIV-1 to human leukocyte antigen class I. Nature 458:641–645. doi:10.1038/nature07746. 19242411 PMC3148020

[15] Leslie AJ, Pfafferott KJ, Chetty P, Draenert R, Addo MM, Feeney M, Tang Y, Holmes EC, Allen T, Prado JG, Altfeld M, Brander C, Dixon C, Ramduth D, Jeena P, Thomas SA, St John A, Roach TA, Kupfer B, Luzzi G, Edwards A, Taylor G, Lyall H, Tudor-Williams G, Novelli V, Martinez-Picado J, Kiepiela P, Walker BD, Goulder PJR 2004 HIV evolution: CTL escape mutation and reversion after transmission. Nat Med 10:282–289. doi:10.1038/nm992. 14770175

[16] Murakoshi H, Koyanagi M, Chikata T, Rahman MA, Kuse N, Sakai K, Gatanaga H, Oka S, Takiguchi M 2017 Accumulation of Pol mutations selected by HLA-B*52:01-C*12:02 protective haplotype-restricted cytotoxic T lymphocytes causes low plasma viral load due to low viral fitness of mutant viruses. J Virol 91:e02082-16. doi:10.1128/JVI.02082-16. 27903797 PMC5286884

[17] Goulder PJR, Watkins DI 2004 HIV and SIV CTL escape: implications for vaccine design. Nat Rev Immunol 4:630–640. doi:10.1038/nri1417. 15286729

[18] Sun X, Shi Y, Akahoshi T, Fujiwara M, Gatanaga H, Schönbach C, Kuse N, Appay V, Gao GF, Oka S, Takiguchi M 2016 Effects of a single escape mutation on T cell and HIV-1 co-adaptation. Cell Rep 15:2279–2291. doi:10.1016/j.celrep.2016.05.017. 27239036

[19] Akahoshi T, Chikata T, Tamura Y, Gatanaga H, Oka S, Takiguchi M 2012 Selection and accumulation of an HIV-1 escape mutant by three types of HIV-1-specific cytotoxic T lymphocytes recognizing wild-type and/or escape mutant epitopes. J Virol 86:1971–1981. doi:10.1128/JVI.06470-11. 22156528 PMC3302409

[20] Allen TM, Yu XG, Kalife ET, Reyor LL, Lichterfeld M, John M, Cheng M, Allgaier RL, Mui S, Frahm N, Alter G, Brown NV, Johnston MN, Rosenberg ES, Mallal SA, Brander C, Walker BD, Altfeld M 2005 De novo generation of escape variant-specific CD8+ T-cell responses following cytotoxic T-lymphocyte escape in chronic human immunodeficiency virus type 1 infection. J Virol 79:12952–12960. doi:10.1128/JVI.79.20.12952-12960.2005. 16188997 PMC1235830

[21] Feeney ME, Tang Y, Pfafferott K, Roosevelt KA, Draenert R, Trocha A, Yu XG, Verrill C, Allen T, Moore C, Mallal S, Burchett S, McIntosh K, Pelton SI, St John MA, Hazra R, Klenerman P, Altfeld M, Walker BD, Goulder PJR 2005 HIV-1 viral escape in infancy followed by emergence of a variant-specific CTL response. J Immunol 174:7524–7530. doi:10.4049/jimmunol.174.12.7524. 15944251

[22] Carlson JM, Du VY, Pfeifer N, Bansal A, Tan VYF, Power K, Brumme CJ, Kreimer A, DeZiel CE, Fusi N, Schaefer M, Brockman MA, Gilmour J, Price MA, Kilembe W, Haubrich R, John M, Mallal S, Shapiro R, Frater J, Harrigan PR, Ndung'u T, Allen S, Heckerman D, Sidney J, Allen TM, Goulder PJR, Brumme ZL, Hunter E, Goepfert PA 2016 Impact of pre-adapted HIV transmission. Nat Med 22:606–613. doi:10.1038/nm.4100. 27183217 PMC4899163

[23] Qin K, Boppana S, Du VY, Carlson JM, Yue L, Dilernia DA, Hunter E, Mailliard RB, Mallal SA, Bansal A, Goepfert PA 2019 CD8 T cells targeting adapted epitopes in chronic HIV infection promote dendritic cell maturation and CD4 T cell trans-infection. PLoS Pathog 15:e1007970. doi:10.1371/journal.ppat.1007970. 31398241 PMC6703693

[24] Fujiwara M, Tanuma J, Koizumi H, Kawashima Y, Honda K, Mastuoka-Aizawa S, Dohki S, Oka S, Takiguchi M 2008 Different abilities of escape mutant-specific cytotoxic T cells to suppress replication of escape mutant and wild-type human immunodeficiency virus type 1 in new hosts. J Virol 82:138–147. doi:10.1128/JVI.01452-07. 17959671 PMC2224353

[25] Pohlmeyer CW, Buckheit RW, Siliciano RF, Blankson JN 2013 CD8+ T cells from HLA-B*57 elite suppressors effectively suppress replication of HIV-1 escape mutants. Retrovirology 10:152. doi:10.1186/1742-4690-10-152. 24330837 PMC3878989

[26] Kaslow RA, Carrington M, Apple R, Park L, Muñoz A, Saah AJ, Goedert JJ, Winkler C, O’Brien SJ, Rinaldo C, Detels R, Blattner W, Phair J, Erlich H, Mann DL 1996 Influence of combinations of human major histocompatibility complex genes on the course of HIV-1 infection. Nat Med 2:405–411. doi:10.1038/nm0496-405. 8597949

[27] O’Brien SJ, Gao X, Carrington M 2001 HLA and AIDS: a cautionary tale. Trends Mol Med 7:379–381. doi:10.1016/S1471-4914(01)02131-1. 11530315

[28] Carrington M, O’Brien SJ 2003 The influence of HLA genotype on AIDS. Annu Rev Med 54:535–551. doi:10.1146/annurev.med.54.101601.152346. 12525683

[29] Trachtenberg E, Korber B, Sollars C, Kepler TB, Hraber PT, Hayes E, Funkhouser R, Fugate M, Theiler J, Hsu YS, Kunstman K, Wu S, Phair J, Erlich H, Wolinsky S 2003 Advantage of rare HLA supertype in HIV disease progression. Nat Med 9:928–935. doi:10.1038/nm893. 12819779

[30] Kiepiela P, Leslie AJ, Honeyborne I, Ramduth D, Thobakgale C, Chetty S, Rathnavalu P, Moore C, Pfafferott KJ, Hilton L, Zimbwa P, Moore S, Allen T, Brander C, Addo MM, Altfeld M, James I, Mallal S, Bunce M, Barber LD, Szinger J, Day C, Klenerman P, Mullins J, Korber B, Coovadia HM, Walker BD, Goulder PJ 2004 Dominant influence of HLA-B in mediating the potential co-evolution of HIV and HLA. Nature 432:769–775. doi:10.1038/nature03113. 15592417

[31] Naruto T, Gatanaga H, Nelson G, Sakai K, Carrington M, Oka S, Takiguchi M 2012 HLA class I-mediated control of HIV-1 in the Japanese population, in which the protective HLA-B*57 and HLA-B*27 alleles are absent. J Virol 86:10870–10872. doi:10.1128/JVI.00689-12. 22811530 PMC3457252

[32] Chikata T, Tran GV, Murakoshi H, Akahoshi T, Qi Y, Naranbhai V, Kuse N, Tamura Y, Koyanagi M, Sakai S, Nguyen DH, Nguyen DT, Nguyen HT, Nguyen TV, Oka S, Martin MP, Carrington M, Sakai K, Nguyen KV, Takiguchi M 2018 HLA class I-mediated HIV-1 control in Vietnamese infected with HIV-1 subtype A/E. J Virol 92:e01749-17. doi:10.1128/JVI.01749-17. 29237835 PMC5809730

[33] Fellay J, Shianna KV, Ge D, Colombo S, Ledergerber B, Weale M, Zhang K, Gumbs C, Castagna A, Cossarizza A, Cozzi-Lepri A, De Luca A, Easterbrook P, Francioli P, Mallal S, Martinez-Picado J, Miro JM, Obel N, Smith JP, Wyniger J, Descombes P, Antonarakis SE, Letvin NL, McMichael AJ, Haynes BF, Telenti A, Goldstein DB 2007 A whole-genome association study of major determinants for host control of HIV-1. Science 317:944–947. doi:10.1126/science.1143767. 17641165 PMC1991296

[34] Carrington M, Nelson GW, Martin MP, Kissner T, Vlahov D, Goedert JJ, Kaslow R, Buchbinder S, Hoots K, O’Brien SJ 1999 HLA and HIV-1: heterozygote advantage and B*35-Cw*04 disadvantage. Science 283:1748–1752. doi:10.1126/science.283.5408.1748. 10073943

[35] Costello C, Tang J, Rivers C, Karita E, Meizen-Derr J, Allen S, Kaslow RA 1999 HLA-B*5703 independently associated with slower HIV-1 disease progression in Rwandan women. AIDS 13:1990–1991. doi:10.1097/00002030-199910010-00031. 10513667

[36] Migueles SA, Sabbaghian MS, Shupert WL, Bettinotti MP, Marincola FM, Martino L, Hallahan CW, Selig SM, Schwartz D, Sullivan J, Connors M 2000 HLA B*5701 is highly associated with restriction of virus replication in a subgroup of HIV-infected long term nonprogressors. Proc Natl Acad Sci U S A 97:2709–2714. doi:10.1073/pnas.050567397. 10694578 PMC15994

[37] Murakoshi H, Koyanagi M, Akahoshi T, Chikata T, Kuse N, Gatanaga H, Rowland-Jones SL, Oka S, Takiguchi M 2018 Impact of a single HLA-A*24:02-associated escape mutation on the detrimental effect of HLA-B*35:01 in HIV-1 control. EBioMedicine 36:103–112. doi:10.1016/j.ebiom.2018.09.022. 30249546 PMC6197679

[38] Juarez-Molina CI, Valenzuela-Ponce H, Avila-Rios S, Garrido-Rodriguez D, Garcia-Tellez T, Soto-Nava M, Garcia-Morales C, Goulder P, Reyes-Teran G 2014 Impact of HLA-B*35 subtype differences on HIV disease outcome in Mexico. AIDS 28:1687–1690. doi:10.1097/QAD.0000000000000322. 24853499

[39] Goulder PJR, Walker BD 2012 HIV and HLA class I: an evolving relationship. Immunity 37:426–440. doi:10.1016/j.immuni.2012.09.005. 22999948 PMC3966573

[40] Pereyra F, Jia X, McLaren PJ, Telenti A, de Bakker PIW, Walker BD, Ripke S, Brumme CJ, Pulit SL, Carrington M, Kadie CM, Carlson JM, Heckerman D, Graham RR, Plenge RM, Deeks SG, Gianniny L, Crawford G, Sullivan J, Gonzalez E, Davies L, Camargo A, Moore JM, Beattie N, Gupta S, Crenshaw A, Burtt NP, Guiducci C, Gupta N, Gao X, Qi Y, Yuki Y, Piechocka-Trocha A, Cutrell E, Rosenberg R, Moss KL, Lemay P, O’Leary J, Schaefer T, Verma P, Toth I, Block B, Baker B, Rothchild A, Lian J, Proudfoot J, Alvino DML, Vine S, Addo MM, Allen TM, 2010 The major genetic determinants of HIV-1 control affect HLA class I peptide presentation. Science 330:1551–1557. doi:10.1126/science.1195271. 21051598 PMC3235490

[41] Itoh Y, Mizuki N, Shimada T, Azuma F, Itakura M, Kashiwase K, Kikkawa E, Kulski JK, Satake M, Inoko H 2005 High-throughput DNA typing of HLA-A, -B, -C, and -DRB1 loci by a PCR-SSOP-Luminex method in the Japanese population. Immunogenetics 57:717–729. doi:10.1007/s00251-005-0048-3. 16215732

[42] Teixeira SLM, de Sá NBR, Campos DP, Coelho AB, Guimarães ML, Leite TCNF, Veloso VG, Morgado MG 2014 Association of the HLA-B*52 allele with non-progression to AIDS in Brazilian HIV-1-infected individuals. Genes Immun 15:256–262. doi:10.1038/gene.2014.14. 24718028

[43] Saito S, Ota S, Yamada E, Inoko H, Ota M 2000 Allele frequencies and haplotypic associations defined by allelic DNA typing at HLA class I and class II loci in the Japanese population. Tissue Antigens 56:522–529. doi:10.1034/j.1399-0039.2000.560606.x. 11169242

[44] Chikata T, Murakoshi H, Koyanagi M, Honda K, Gatanaga H, Oka S, Takiguchi M 2017 Control of HIV-1 by an HLA-B*52:01-C*12:02 protective haplotype. J Infect Dis 216:1415–1424. doi:10.1093/infdis/jix483. 28968792

[45] Murakoshi H, Kuse N, Akahoshi T, Zhang Y, Chikata T, Borghan MA, Gatanaga H, Oka S, Sakai K, Takiguchi M 2019 Broad recognition of circulating HIV-1 by HIV-1-specific cytotoxic T-lymphocytes with strong ability to suppress HIV-1 replication. J Virol 93:e01480-18. doi:10.1128/JVI.01480-18. 30333175 PMC6288335

[46] Chikata T, Carlson JM, Tamura Y, Borghan MA, Naruto T, Hashimoto M, Murakoshi H, Le AQ, Mallal S, John M, Gatanaga H, Oka S, Brumme ZL, Takiguchi M 2014 Host-specific adaptation of HIV-1 subtype B in the Japanese population. J Virol 88:4764–4775. doi:10.1128/JVI.00147-14. 24522911 PMC3993807

[47] Falk K, Rötzschke O, Takiguchi M, Gnau V, Stevanović S, Jung G, Rammensee HG 1995 Peptide motifs of HLA-B51, -B52 and -B78 molecules, and implications for Behćet’s disease. Int Immunol 7:223–228. doi:10.1093/intimm/7.2.223. 7734418

[48] Yagita Y, Kuse N, Kuroki K, Gatanaga H, Carlson JM, Chikata T, Brumme ZL, Murakoshi H, Akahoshi T, Pfeifer N, Mallal S, John M, Ose T, Matsubara H, Kanda R, Fukunaga Y, Honda K, Kawashima Y, Ariumi Y, Oka S, Maenaka K, Takiguchi M 2013 Distinct HIV-1 escape patterns selected by cytotoxic T cells with identical epitope specificity. J Virol 87:2253–2263. doi:10.1128/JVI.02572-12. 23236061 PMC3571484

[49] Zou C, Murakoshi H, Kuse N, Akahoshi T, Chikata T, Gatanaga H, Oka S, Hanke T, Takiguchi M 2019 Effective suppression of HIV-1 replication by cytotoxic T lymphocytes specific for Pol epitopes in conserved mosaic vaccine immunogens. J Virol 93:e02142-18. doi:10.1128/JVI.02142-18. 30674626 PMC6430542

[50] Altman JD, Moss PA, Goulder PJ, Barouch DH, McHeyzer-Williams MG, Bell JI, McMichael AJ, Davis MM 1996 Phenotypic analysis of antigen-specific T lymphocytes. Science 274:94–96. doi:10.1126/science.274.5284.94. 8810254

[51] Kuse N, Sun X, Akahoshi T, Lissina A, Yamamoto T, Appay V, Takiguchi M 2019 Priming of HIV-1-specific CD8 T cells with strong functional properties from naïve T cells. EBioMedicine 42:109–119. doi:10.1016/j.ebiom.2019.03.078. 30956171 PMC6491959

[52] Schönbach C, Ibe M, Shiga H, Takamiya Y, Miwa K, Nokihara K, Takiguchi M 1995 Fine tuning of peptide binding to HLA-B*3501 molecules by nonanchor residues. J Immunol 154:5951–5958. 7751638

[53] Takamiya Y, Schönbach C, Nokihara K, Yamaguchi M, Ferrone S, Kano K, Egawa K, Takiguchi M 1994 HLA-B*3501-peptide interactions: role of anchor residues of peptides in their binding to HLA-B*3501 molecules. Int Immunol 6:255–261. doi:10.1093/intimm/6.2.255. 8155602

[54] Tanabe M, Sekimata M, Ferrone S, Takiguchi M 1992 Structural and functional analysis of monomorphic determinants recognized by monoclonal antibodies reacting with the HLA class I alpha 3 domain. J Immunol 148:3202–3209. 1374450

[55] Tomiyama H, Akari H, Adachi A, Takiguchi M 2002 Different effects of Nef-mediated HLA class I down-regulation on human immunodeficiency virus type 1-specific CD8(+) T-cell cytolytic activity and cytokine production. J Virol 76:7535–7543. doi:10.1128/jvi.76.15.7535-7543.2002. 12097566 PMC136399

